# Letter from the Editor-in-Chief

**DOI:** 10.19102/icrm.2017.080606

**Published:** 2017-06-15

**Authors:** Moussa Mansour


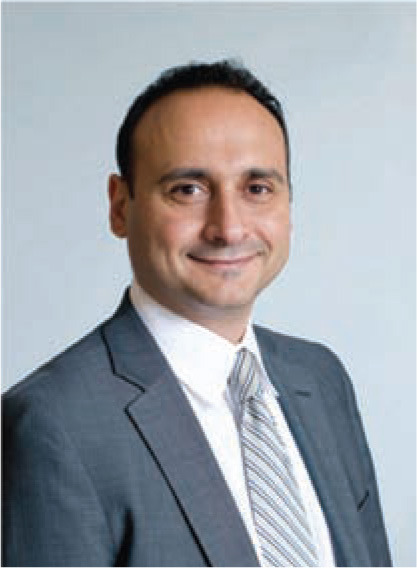


Dear Readers,

In the last two years, there has been an increased interest in using objective markers for the assessment of adequate radiofrequency (RF) ablation lesion formation. The growth of this trend has been especially propelled by the introduction of contact force sensing, which is now standard practice in most electrophysiology laboratories that use RF energy. The article by Boles et al. in this issue of *The Journal of Innovations in Cardiac Rhythm Management* is one such exemplar, during which the authors describe their experience with the use of the lesion size index (LSI) parameter in an ablation of typical atrial flutter. In the study, 15 patients with atrial flutter underwent cavotricuspid isthmus ablation guided by LSI, and were compared to 23 patients who had conventional ablation. The use of an LSI of 5-6, a force-time integral (FTI) of 500-600 gs, and a contact force value of more than 10 g, lead to the desired outcome in all subjects.

This study enrolled a small number of patients and thus, no meaningful conclusions about the safety and efficacy of this approach can be derived from it. However, the importance of this publication rests in its discussion of the use of objective markers for assessing adequate lesion formation. Not so long ago, operators had very limited markers to rely upon for adequate lesion formation, including electrogram amplitude reduction and impedance drop. More recently, however, studies have demonstrated the limitations of using these markers, which eventually led to the introduction of indices that combine information from many inputs. The first index used was FTI, a simple product of force and time. Other indices were subsequently introduced including LSI, which combines information from power, time, and contact force. Even more complex indices are currently being tested, and combine information from additional inputs including catheter stability, impedance drop, catheter-tissue interface temperature, and others. While these indices are “surrogates” for the assessment of adequate lesion formation, they have been found to provide an accurate estimation of RF lesion size. In fact, with the use of some of these indices, lesion depth can be estimated with an error of less than 1 mm.

In our practice at Massachusetts General Hospital in Boston, we rely heavily on LSI and FTI to guide ablations. For LSI, we believe that a value around 5 is a marker of adequate lesion formation in the atria. However extreme caution should be exercised when ablating in the posterior wall of the left atrium, as it is a place where RF should be delivered at low power, duration, and contact force, regardless of the resulting LSI or FTI.

I hope that you enjoy reading the above-mentioned article and other interesting publications in this issue of *The Journal of Innovations in Cardiac Rhythm Management.*

Sincerely,


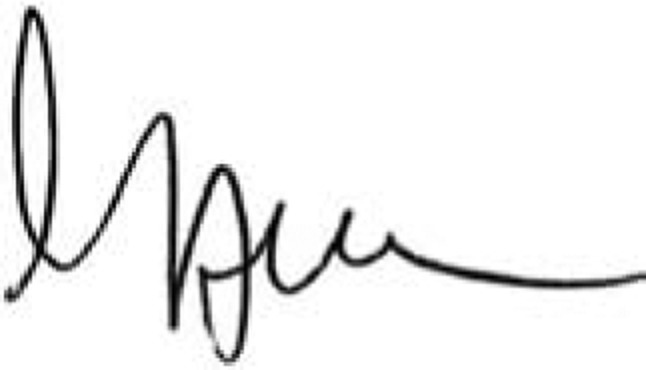


Moussa Mansour, MD, FHRS, FACC

Editor-in-Chief

The Journal of Innovations in Cardiac Rhythm Management

MMansour@InnovationsInCRM.com

Director, Atrial Fibrillation Program

Massachusetts General Hospital

Boston, MA 02114

